# Evaluation of the Prebiotic Potential of a Commercial Synbiotic Food Ingredient on Gut Microbiota in an Ex Vivo Model of the Human Colon

**DOI:** 10.3390/nu12092669

**Published:** 2020-09-01

**Authors:** Walid Mottawea, Salma Sultan, Kara Landau, Nicolas Bordenave, Riadh Hammami

**Affiliations:** 1 School of Nutrition Sciences, Faculty of Health Sciences, University of Ottawa, Ottawa, ON K1N 6N5, Canada; wmott020@uottawa.ca (W.M.); ssult017@uottawa.ca (S.S.); Nicolas.Bordenave@uottawa.ca (N.B.); 2 Department of Microbiology and Immunology, Faculty of Pharmacy, Mansoura University, Mansoura 35516, Egypt; 3 Uplift Food Pty Ltd., New York, NY 10001, USA; klandau@upliftfood.com; 4 School of Chemistry and Biomolecular Sciences, Faculty of Sciences, University of Ottawa, Ottawa, ON K1N 6N5, Canada

**Keywords:** gut microbiota, prebiotics, synbiotics, psycho-biotics, microbiota-gut-brain axis

## Abstract

Behavior and mood disorders have been linked to gut microbiota dysbiosis through the “microbiota-gut-brain axis”. Microbiota-targeting interventions are promising therapeutic modalities to restore or even maintain normal microbiome composition and activity in these disorders. Here, we test the impact of a commercial synbiotic formulation on gut microbiota composition and metabolic activity. We employed an ex-vivo continuous fermentation model that simulates the proximal colon to assess the effect of this formulation on microbiota structure and functionality as compared to no treatment control and microcrystalline cellulose as a dietary fiber control. The test formulation did not alter the diversity of gut microbiota over 48 h of treatment. However, it induced the enrichment of *Lactobacillus*, *Collinsella* and *Erysipelotrichaceae*. The test formulation significantly increased the level of microbiota-generated butyrate within 12 h of treatment as compared to 24 h required by microcrystalline cellulose to boost its production. The test formulation did not lead to a significant change in amino acid profiles. These results provide evidence of potential benefits related to synbiotic effects and general gut health and support the potential of this food formulation as a therapeutic dietary intervention in mood and behavior disorders.

## 1. Introduction

There is growing evidence that gut microbiota dysbiosis is intimately linked with behavior and mood disorders such as autism spectrum disorder (ASD), anxiety and depression [1]. For example, the microbiota of children with ASD have been shown to have lower fermentative bacteria, such as *Prevotella copri*, and exhibit a lower overall diversity [2]. This link has been referred to as the “gut-brain axis” - the connection of gut microbiota to the host central nervous system via neuronal, immunological and endocrinal connections [3]. It has been suggested that this interaction can be directly mediated through microbial metabolites such as neurotransmitters, short-chain fatty acids (SCFAs) or vitamins, or indirectly through endocrine, immune or metabolic systems [4,5]. For example, in major depressive disorder, the brain signatures associated with depression have been negatively correlated with the relative abundance of *Bacteroides*, which has the capacity to generate the neurotransmitter γ-aminobutyric acid (GABA) [5].

To date, therapeutic interventions targeting microbiota include mostly dietary intervention and microbiota transfer therapy (MTT). MTT has shown promising results, for example towards the improvement of ASD symptoms [1], but its efficiency as biotherapy is still controversial [6], with many limitations including resistance to colonization, adverse effects, donor selection, sample handling, route of administration and cost-effectiveness [7]. Conversely, dietary interventions, including the administration of probiotics, prebiotics, and synbiotics, hold a particular appeal for their safety and non-invasive character as well as their cost-effectiveness [8]. They have showed success as microbiota management tools in various diseases [9,10]. For example, the intake of *B. coagulans* has been reported to increase the levels of fecal *F. prausnitzii* and SCFAs [11]. *F. prausnitzii* and butyrate are associated with antidepressive properties [12]. Also, *Bacillus coagulans*, *Faecalibacterium prausnitzii*, *Lactobacillus helveticus* and *Bifidobacterium longum* were reported to reduce anxiety-like behavior in animals and demonstrated positive psychological effects and decreased serum cortisol levels in humans [12,13,14,15]. Prebiotics-based manipulation of gut microbiota has also shown promising beneficial effects on behavior and mood in animal models and humans. For instance, the administration of B-GOS^®^ to neonatal rats prior to weaning led to increased levels of brain proteins such as BDNF, synaptophysin, and the GluN2A subunit in the hippocampus [16], indicating the influence of the gut microbiome on brain development. Consumption of B-GOS^®^ has also been shown to lower waking cortisol levels and improve emotional bias in healthy human participants [17].

Therefore, although the neurochemical mechanisms by which psycho-biotics manipulate the microbiota-gut-brain axis are yet to be fully understood [18,19], there is growing interest in developing food products with probiotics and/or prebiotics that could hold the potential for positive psychological effects. Considering the putative mechanisms of action of psycho-biotics, such food products or preliminary formulations could be screened ex vivo for their ability to increase microbiota diversity, increase microbial production of bioactive metabolites such as SCFA, neurotransmitters GABA, serotonin and dopamine, and for their ability to maintain a population of microbiota of interest that have been linked to positive psychological outcomes. Promising candidates could then be evaluated in vivo through clinical trials in humans. Indeed, ex vivo proximal colon models are an efficient approach to test the response of gut microbiota to dietary interventions reducing biases found in animal studies due to variable dietary intakes by the animal groups themselves due to differences in palatability or satiety regulation [20,21,22].

In the current study, we aim to assess the potential of a commercial synbiotic formulation to modulate gut microbiota composition and functionality in relation to potential psycho-biotic activity. This formulation includes lupin flour (containing mainly cellulose, galactan and galactomannan polysaccharides) [23], tapioca fiber (containing mainly resistant dextrin) [24], tiger-nut flour (containing mainly xyloglucan polysaccharides and rich in flavonoids and sterols such as quercetin, myricetin and stigmasterol) [25,26], gold kiwifruit powder (containing mainly pectin polysaccharides and rich in flavonoids) [27] and probiotic *Bacillus coagulans* GBl-30,6086 (GanedenBC30) [28]. We employed an ex vivo continuous fermentation model that mimics the physiological conditions of the proximal colon to test this formulation against a no-treatment control and a control consisting of common, non-fermentative, food-grade dietary fiber (microcrystalline cellulose). Microbiota composition and diversity were monitored with 16S-rRNA amplicon sequencing with specific attention to *B. coagulans* and *F. Prausnitzii*, and their functionality was assessed by quantification of SCFA, amino acids, GABA, serotonin, and dopamine. As GABA, serotonin, and dopamine are generated by multiple species, we aimed to test the metabolites themselves using Gas Chromatography–Mass Spectrometry (GC/MS). Although the supplemented formula is enriched with these specific amino acids, the fermentation media itself include them.

## 2. Materials and Methods

### 2.1. Study Design and Preparation of Test Samples

The experimental design consisted of a parallel comparison of the following three conditions in an ex vivo colonic fermentation system: (1) a no-treatment control, (2) a test treatment containing a commercial blend of dietary fibers and a probiotic strain, and (3) a control treatment consisting of microcrystalline cellulose. The test sample used for the test treatment consisted of the following dry weight basis composition: 66.5% lupin flour from Inveja SAS (Martigné-Ferchaud, France), 26.3% tapioca fiber powder from Anderson Advanced Ingredients (Irvine, CA, USA), 6.8% tiger-nut flour from Tradin Organic (Scotts Valley, CA, USA), 0.3% (80 × 10^6^ colony forming unit: CFU) probiotic powder ingredient of *Bacillus coagulans* GBl-30,6086 (GanedenBC30) from Kerry Inc. (Beloit, WI, USA) and 0.07% gold kiwifruit powder from Anagenix (Wellington, New-Zealand). Lupin flour, Tapioca fibers, tiger-nut flour and gold kiwifruit powders were mixed and heated at 350 °F for 3 min to mimic food processing, 1.78 gm were hydrated in 5 mL deionized sterile water for 24 h at 4 °C and the probiotic powder was added to the suspension immediately prior to inoculating the bioreactor. The cellulose microcrystalline control underwent the same preparation process before addition to the ex vivo colonic fermentation system.

### 2.2. Fecal Inoculum and Immobilization

The fermentation system was designed to mimic the colonic microbial composition of healthy adults. Therefore, fecal samples of two healthy adults (male; age 39 and female; age 36) were used to inoculate gellan (2.5% *w*/*v*) and xanthan (0.25% *w*/*v*) beads under anaerobic conditions as described previously [29]. Collection of samples was approved by The University of Ottawa Research Ethics Board (certificate H-02-18-347; 29/07/2019) and complied with the following exclusion criteria: donors had not used any antibiotic, prebiotic or probiotic over the two months prior fecal collection, their body mass index was lower than the 95th percentile for age, they had not been diagnosed with diabetes mellitus, and they had not been diagnosed with infectious gastroenteritis over the two months prior to fecal collection.

### 2.3. Experimental Setup and Fermentation Procedures

**Figure 1:**
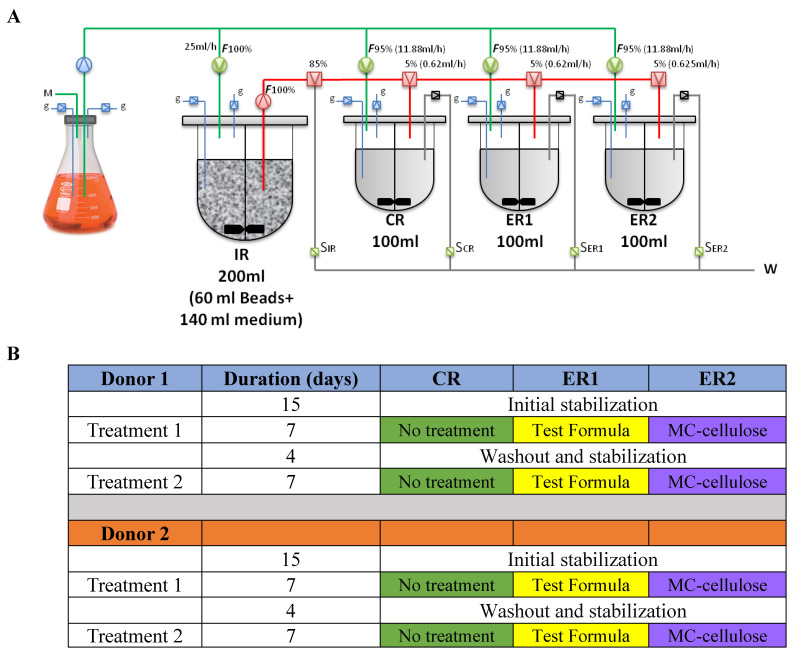
Experimental set up (**A**) and timeline (**B**) of the employed ex vivo colon continuous fermentation model. The gut microbiota was propagated in the inoculating reactor (IR) for 15 days before seeding three sub-reactors (CR no treatment control, ER1 with microcrystalline cellulose control, and ER2 with test sample). M; Nutritive medium, g; pH and dissolved oxygen sensors, W; waste, F; percentage of the medium input (green lines) or inoculum input (red lines).

The microbiota community from healthy donors will be inserted into an ex vivo model mimicking the large intestine (N*u*GUT Research Platform, University of Ottawa). Fermentation simulating physiological and microbiological conditions of the human intestine was described previously [29,30]. Briefly, 60 mL gel beads inoculated a 1 L BioFlo^®^ 120 vessel (Eppendorf, Mississauga, ON, Canada) containing 140 mL Macfarlane culture medium prepared as previously described [11]. A batch fermentation protocol was followed for the first 48 h by replacing 100 mL of the bioreactor medium with a fresh nutrition medium every 12 h. After 48 h, the fermentation was switched to a continuous mode for the rest of the experiment. After 15 days of continuous fermentation, which was previously identified as the required time for microbiota stabilization (Supplementary Materials Figure S1A), the stabilized microbial community in the inoculating reactor (IR) was used for parallel inoculation of 3 DASGIP^®^ bioreactors (Eppendorf, Mississauga, ON) labeled CR (Control Reactor), ER1 (Experimental Reactor 1), and ER2 (Experimental Reactor 2) as shown in Figure 1. The operation conditions were set to pH 5.7, 37 °C, 120 rpm stirring and 8 h retention time. The anaerobic environment was achieved by headspace flushing of N_2_ and CO_2_ at a 0.9:0.1 ratio. The total volume in each sub-reactor was maintained at 100 mL with fresh medium and IR inlets at respective rates of 11.88 and 0.62 mL/h and waste outlet at a rate of 12.5 mL/h, simulating a retention time of 8 h. The three sub-reactors were run without any treatment for 48 h to reach the stability of the microbial community.

Once microbiota stability was achieved in each reactor, the test sample was added to the ER1 bioreactor, the microcrystalline cellulose control sample was added to the ER2 bioreactor, and the CR bioreactor was used as a no-treatment control. The test and cellulose control samples were added to ER1 and ER2 according to the following procedure. 1.78 g of cellulose control and test samples in 5 mL sterile deionized water were added every 12 h to ER1 and ER2 respectively, simulating a total daily intake of 40 g of cookies divided into two servings of 20 g each. Samples of 2 mL were collected from the bioreactors at 0, 4, 8, 12, 24, 36 and 48 h of treatment. The collected samples were centrifuged at 14,000× *g* for 5 min. The pellet was used for metagenomic DNA extraction, while the supernatant was used for metabolite analysis. This fermentation experiment was conducted in duplicate for each fecal sample donor.

### 2.4. Genomic Analyses

#### 2.4.1. DNA Extraction

Metagenomic DNA was extracted using a Fast DNA Spin Kit (MP Biomedicals; Solon, OH, USA) and a Bead Mill-24 Homogenizer (Fisher Scientific; Ottawa, ON, Canada). The protocol was modified as described earlier [31,32,33] to two cycles of mechanical lysis at speed 6.0 m∙s^−1^ for 40 s, with 5 min cooling on ice between the two cycles. Next, the DNA was isolated and purified as per the Fast DNA Spin Kit protocol. The extracted DNA was quantified with a Qubit fluorometer (Invitrogen; Carlsbad, CA, USA) and stored at −20 °C until used for further analysis.

#### 2.4.2. MiSeq Sequencing Analysis

The V3-V4 regions of the 16S rRNA gene were amplified using dual-barcoded primers, and the amplicon library for sequencing was constructed using Illumina standard protocol. The amplicon libraries were pooled, and paired end sequenced with Illumina MiSeq platform (N*u*GUT Research Platform, University of Ottawa) using 600 bp MiSeq Reagent Kit v3 (Illumina; San Diego, CA, USA) as per standard protocol. To control for contaminants, a template-free control and extraction kit reagents control were co-sequenced with the specimens.

#### 2.4.3. qPCR Analysis

The quantification of *Bacillus coagulans* and *Faecalibacterium prausnitzii* relative to total bacteria was determined by qPCR on the extracted metagenomic DNA using a Bio-rad CFX96 Real-Time PCR Detection System and the following specific primers: forward primer BACO186F (5′-GCATGGAGGAAAAAGGAA-3′) and reverse primer BACO447R (5′-CCCGGCAACAGAGTTTTA-3′) for *Bacillus coagulans* [34]; forward primer FaePrF (5′-CCCTTCAGTGCCGCAGT-3′) and reverse primer FaePrR (5′-GTCGCAGGATGTCAAGAC-3′) for *Faecalibacterium prausnitzii* [35]. Each sample was tested in duplicate in a total volume of 20 μL per reaction. 25 ng of template DNA was added to a reaction mixture containing 0.25 μM of each primer and 1× SsoAdvanced Universal SYBR Green Supermix (Bio-Rad; Mississauga, ON, Canada). The amplification conditions were 3 min at 98 °C followed by 40 cycles of 95 °C for 15 s and 60 °C for 30 s with data collection at the second step of each cycle. Ct values were then extracted using Bio-Rad CFX Maestro software, and the relative abundance of each taxon was calculated as a ΔCt value (taxon Ct–universal 16S rRNA Ct) as described earlier [31].

### 2.5. Metabolite Analyses

#### 2.5.1. Quantification of Short Chain Fatty Acids (SCFAs)

Concentrations of short-chain fatty acids (SCFAs), including acetic, propionic, and butyric acids were determined using Gas Chromatography with a Flame Ionization Detector (GC-FID) as described earlier [36]. The peaks were identified and quantified with standards from MilliporeSigma (Oakville, ON, Canada). Results were expressed as the concentration of SCFAs in mM. All samples were analyzed in duplicates (two technical measures).

#### 2.5.2. Quantification of Amino Acids and Neuroactive Metabolites

Amino acid metabolites, GABA, serotonin, and dopamine were quantified using GC coupled with Mass Spectrometry detection (GC-MS). The samples were derivatized with an EZ-faast kit from Phenomenex (Torrance, CA, USA) as per standard protocol using 100 µL of the centrifuged and filtered supernatant. Norvaline (0.2 mM) was used as an internal standard. 1 µL of the derivatized sample was injected onto a 7820A GC-MS system equipped with a DB-1 column (30 m × 0.320 mm × 0.50 µm) both from Agilent Technologies, Inc. (Mississauga, ON, Canada) through a temperature gradient from 80 °C to 300 °C at a rate of 20 °C/min. The compounds were identified and quantified with the amino acid standards provided with the EZ-faast kit. Results are presented as mean ± SEM of four biological replicates.

### 2.6. Metagenomic Sequencing Data Analysis

Sequences were quality filtered and clustered into operational taxonomic units (OTU) based on 97%-similarity using closed-reference OTU picking against Greengenes database (v13.8) from Second Genome Inc. (South San Francisco, CA, USA) as the reference database via QIIME 1.9.0 software [37]. Contaminant OTU were identified and removed if their mean abundance in negative and extraction reagents controls was ≥25% of their mean abundance in the samples. Singleton and doubleton OTU and OTU that occurred in less than 10% of the samples were removed. The remaining OTU were rarefied into an equal number of 18,000 reads per sample using QIIME. Alpha diversity was estimated with Chao1 estimate and Shannon index. Beta diversity among samples was calculated using Bray-Curtis distance and visualized using Principal Coordinate Analysis (PCoA). The contribution of different treatments to the diversity of gut microbiota community was assessed from the Bray-Curtis distance matrix using the Adonis package for R (R Foundation for Statistical Computing, Vienna, Austria) and 999 permutations [38]. To identify differential taxa among different treatments, linear discriminant effect size analysis was conducted on the relative abundance of different taxa levels [39]. Samples were labeled with the treatment type as the sample class, and the time points as the subclass. Taxa with log_10_ LDA score ≥2 and *p* < 0.05 were considered significant. When required Kruskal-Wallis test was applied for statistical analysis, and P-values were corrected using two-stage Benjamini, Krieger, and Yekutieli false discovery rate (FDR) procedure.

## 3. Results

### 3.1. Diversity of the Gut Microbiota

**Figure 2:**
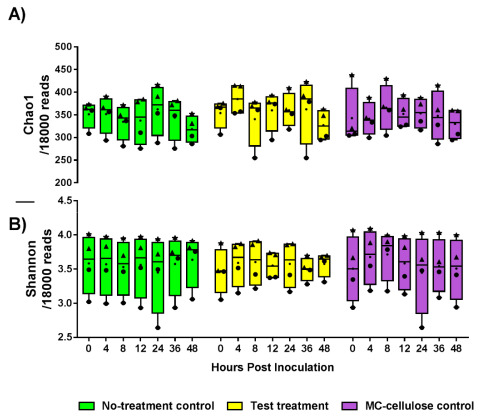
Chao1 estimates (**A**) and Shannon indices (**B**) of the identified microbiota from bioreactors with a no-treatment control, test treatment and microcrystalline cellulose control at different times post-treatment (*n* = four biological replicates each; triangles are for donor 1 samples and circles for donor 2 samples). Results were calculated from rarefied 18,000 reads per sample. Middle lines represent mean. Data were analyzed using the Kruskal-Wallis test and Two-stage Benjamini, Krieger, and Yekutieli FDR procedure (*p* > 0.05).

Alpha-diversity of the microbiota from the three bioreactors between 0 and 48 h was evaluated with Chao1 estimates and Shannon indices of the identified OTU. Results are shown in Figure 2. No significant differences were found, and neither the test treatment nor microcrystalline cellulose control treatment affected the microbiota diversity (*p* > 0.05).

**Figure 3:**
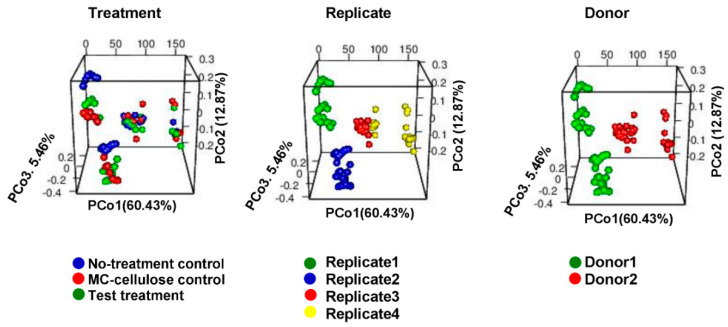
Plots of Principal Coordinate Analysis (PCoA) based on Bray-Curtis distances among the identified microbiota in different samples, showing clustering based on the type of treatment, replicate and donor. The samples were colored as indicated in legends. PCoA1, PCoA2 and PCoA3 represent the top three coordinates that captured the highest microbial variability among samples, and the percentage shown indicates the fraction of variation represented by each coordinate. Adonis analysis was used to test for the statistical significance of sample grouping.

In order to identify which factor controls the microbiota diversity among different samples, beta-diversity across treatments, donor and biological replicates was evaluated by PCoA based on Bray-Curtis distances. Plots of PCoA are shown in Figure 3. The microbiota of different treatments was clustered primarily by donor (Adonis *R*^2^ = 0.56; *p* = 0.001), and by experiment replicate within each donor (Adonis *R*^2^ = 0.736; *p* = 0.001). Moreover, for each experiment replicate, the microbiota was clustered based on treatment (Adonis *R*^2^ = 0.717; *p* = 0.001).

### 3.2. Composition of Gut Microbiota

The composition of gut microbiota was evaluated according to treatment. Overall, an average of 225 OTUs were identified per sample. Six bacteria phyla were identified in the generated dataset including Firmicutes (75.9 ± 0.8%), Bacteroidetes (8.1 ± 0.9%), Proteobacteria (5.8 ± 0.2%), Actinobacteria (10.8 ± 0.5%), Cyanobacteria and Saccharibacteria (formerly TM7) (<0.00001). The microbiota developed from the two fecal sample donors were different at the family level, where donor 1 microbiota developed into a community dominated by *Bacteroidaceae*, *Lachnospiraceae* and *Veillonellaceae*, while the microbial community from the second donor was enriched in *Lachnospiraceae* and *Veillonellaceae* with low abundance of *Bacteroidaceae* (Figures S1B and S2).

**Figure 4:**
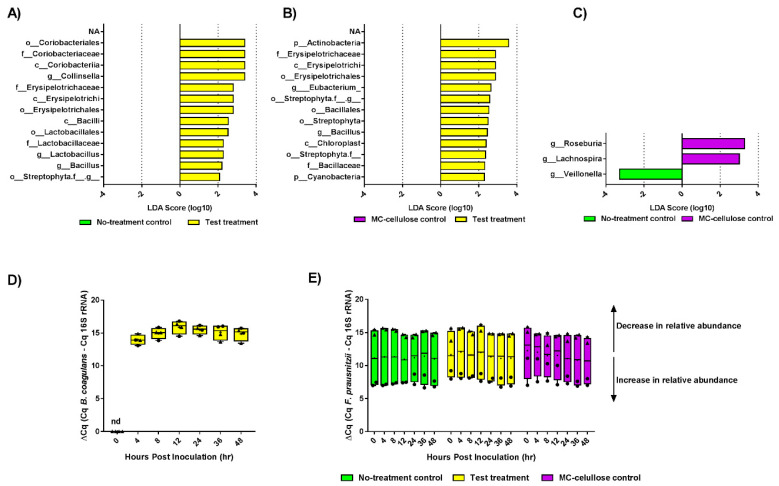
The test formulation modulates gut microbiota composition. (**A**–**C**) Histograms of the linear discriminant analysis (LDA) scores showing microbial taxa that vary significantly in abundance between: (**A**) no-treatment control and test treatment, (**B**) microcrystalline cellulose control and test treatment, (**C**) no-treatment control and microcrystalline cellulose treatment. (**C**,**D**) Relative abundance charts for target species: (**D**) relative abundance of inoculated probiotic, *B. coagulans*, over 48 h with test treatment; (**E**) relative abundance of *F. prausnitzii* over 48 h with no-treatment control, microcrystalline cellulose control treatment and test treatment. Triangle symbols indicate donor 1 samples and circles for donor 2 samples.

Linear discriminate analysis displayed in Figure 4 showed that the test sample led to major changes in microbiota composition in comparison to both the no-treatment control and the microcrystalline cellulose. Indeed, microbiota subjected to the test sample was enriched in *Bacillus*, *Lactobacillus*, and *Collinsella* at the genus level, *Coribacteriaceae*, *Erysipelotrichaceae*, and *Lactobacillaceae* at the family levels and Streptophyta at the order level compared to untreated microbiota (Figure 4A and Figure S3A; *p* < 0.05). Microbiota subjected to the test sample was also enriched in Actinobacteria, Cyanobacteria, *Erysipelotrichaceae*, *Bacillaceae*, chloroplast, Streptophyta and *Eubacterium* (Figure 4B and Figure S3B; *p* < 0.05). In contrast, the microcrystalline cellulose control led to a minor modification of microbiota compared to the no-treatment control, with enrichment of *Roseburia* and *Lachnospira* and depletion of *Veillonella* (Figure 4C; *p* < 0.05).

As the probiotic *Bacillus coagulans* GBI-30, 6086 (GanedenBC30) was included in the test treatment, PCR analysis was used to detect this species and quantify its relative abundance with all treatments. *B. coagulans* was detected only in the microbiota treated with the commercial formulation (test treatment) but neither in microcrystalline cellulose control treatment nor in the microbiota of the no-treatment control. The relative abundance of *B. coagulans* with the test treatment is shown in Figure 4D: the relative abundance of *B. coagulans* increased significantly after 4 h of treatment, reached its maximum after 12 h and remained constant for the following 36 h post-treatment. The intake of *B. coagulans* has been previously reported to increase the fecal levels of *F. prausnitzii* [40], a key gut microbe associated with antidepressive properties [12]. Therefore, the relative abundance of *F. prausnitzii* evaluated by qPCR showed no significant difference after treating the microbiota with either the microcrystalline cellulose control or the test treatment (Figure 4E; *p* > 0.05).

### 3.3. Metabolite Production

**Figure 5:**
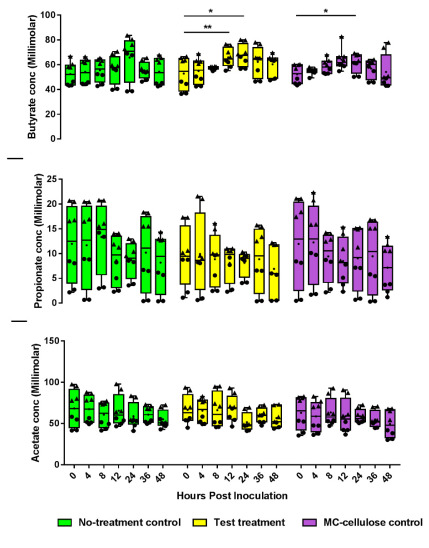
Short-chain fatty acids (SCFAs) concentration measured by GC over 48 h with a no-treatment control, microcrystalline cellulose control, and test treatment: butyrate, propionate and acetate from top to bottom. Each time point is represented with 4 biological replicates × 2 technical measures (triangles are for donor 1 samples and circles for donor 2 samples). Statistical comparisons were conducted among different treatments at the same time and among different time points within each treatment using repeated measures ANOVA test with Bonferroni as a post hoc test for multiple comparisons. Significant differences were established identified only for butyrate using levels generated at different time points (* *p* < 0.05; ** *p* < 0.01).

Production of bioactive metabolites (SCFAs, GABA, dopamine, and serotonin) by the microbiota according to treatment was quantified by GC-MS. We detected the three major SCFAs generated by gut microbiota including: Acetate: Average over 48 h ± SEM of 62.4 ± 1.8, 62.05 ± 27 and 58.15 ± 1.8 mM; Butyrate: 56.29 ± 1.6, 60.17 ± 2 and 57.44 ± 1.5 mM; and Propionate: 10.39 ± 0.75, 8.6 ± 0.37 and 9.6 ± 0.69 Mm in the untreated control, test treatment and microcrystalline cellulose control groups, respectively (Figure 5). Differences were found in microbiota-generated butyrate over time. Whereas butyrate level increased significantly after 24 h under the microcrystalline cellulose treatment (Figure 5; *p* < 0.05), it increased significantly after only 12 h under the test treatment (*p* < 0.01) and remained significantly enriched after 24 h (*p* < 0.05) as shown in Figure 5. Complete amino acid profile analyses were carried out over 48 h for all treatments. Test treatment did not lead to a significant change in amino acid profile compared to controls. Treatments also did not affect the production of GABA, dopamine, and serotonin.

## 4. Discussion

The results of this study show that we succeeded in developing two distinct gut microbiota communities using the ex vivo proximal colon model. We identified an average of 225 OTU per sample, which is comparable to the minimum of 160 species estimated to be harbored by each individual [41]. The identified OTUs originated from six phyla with more than 80% relative abundance of Bacteroidetes and Firmicutes, which is a common structure of the gut microbiota [31,32,42]. The two microbiota communities developed in the ex vivo system were different at the family level. The developed microbial community from donor 1 is dominated by *Bacteroidaceae,* which is a similar characteristic as that of his stool microbiota. Comparatively, donor 2 developed microbiota highly abundant in *Lachnospiraceae,* which is dominant in his stool source, illustrating two distinct enterotypes of the two communities [43,44]. Still, the fermentation medium in our system has a sufficient amount of mucin to induce enrichment of the *Veillonellaceae* family. We still need to consider the variability between the stool and the proximal colon microbiota which we tried to mimic in the fermenter as this is the main part of microbiota saccharolytic fermentation in the gut (Hamer et al., 2012). For instance, *Akkermansia,* which is enriched in the fermented microbiota compared to stool, is most abundantly present in the proximal colon [45]. It is known that stool samples exhibit different profiles of gut microbiota as compared to any other gut segment, including the proximal colon [32,42]. Due to the time and cost constraints, the current ex vivo model is limited by the low number of biological replicates and the short period of treatment. It also does not exhibit the same complexity as the gut mucosal environment, e.g., lacking the immunological and some physiological modulatory factors, which results in enrichment or depletion of some microbial populations [46]. Although, we should be careful about generalizing our findings to overall human gut microbiota, this study illustrates that the test formulation has a potential synbiotic impact on gut microbiota composition and metabolic functionality.

The test treatment did not significantly affect the diversity of the microbiota over 48 h. Although it is has been shown that temporary dietary shifts can change the microbiota structure within days [47], the exact time frame for diet-induced shifts is person- and diet-specific [48] and can require between three and five days depending on the diet composition [47]. Therefore, we can assume that the short duration of the treatment tested in the present study (48 h) limited the ability of the test treatment to change the microbiota structure. Nevertheless, in comparison with untreated microbiota and that treated with microcrystalline cellulose, the test formulation altered the relative abundance of some microbial taxa, which suggests a synbiotic effect of the formulation.

Indeed, we observed enrichment of *Lactobacillus* after the addition of the test formulation. This may be attributed to the flavonoids and Galacto-oligosaccharide (GOS) contents of the test formulation. Flavonoids can be metabolized by multiple *Lactobacillus* spp. [49]. In addition, different species of *Lactobacillus* harbor β-galactosidases that enable them to grow fast on GOS [50]. The abundance of *Lactobacillus* has been reported to directly relate to positive self-judgment, through which it is indirectly related to cognitive depression and reduced cognitive empathy [51]. Enrichment of *Lactobacillus* is associated with lowering Beck Depression Inventory scores, serum insulin level, serum high-sensitivity C-reactive protein, and biomarkers of oxidative stress in patients with major depressive disorder (MDD) [52].

Additionally, the microbiota treated with the test formulation had increased levels of *Collinsella*. *Collinsella aerofaciens* has been associated with a low risk of colon cancer and IBS [53,54]. Different plant prebiotics is known to induce the enrichment of *Collinsella* [55]. The increased levels of *Collinsella* after prebiotic treatment has been shown to be associated with high urinary levels of Hippurate, a gut-derived metabolite commonly associated with a healthy status [56] reduction of intestinal discomfort [57]. Similarly, the *Erysipelotrichaceae* family significantly increased after the addition of the test formulation. *Erysipelotrichaceae* are flavonoid-metabolizing gut microbes [49,58]. In addition, *Erysipelotrichaceae* members are saccharolytic fermenters, their levels being positively correlated with dietary fiber consumption [59,60]. The role of *Erysipelotrichaceae* members in health is controversial. A previous study investigating altered microbiota composition in patients with MDD has reported that the relative proportion of *Erysipelotrichaceae* was significantly lower in the MDD than in the healthy control group [9]. Some studies have reported an association between enrichment of *Erysipelotrichaceae* and disease phenotypes such as Parkinson’s disease, obesity, and inflammation [61,62,63]. In contrast, many studies have shown a lower abundance of that family in inflammatory bowel disease (IBD) cohorts [64,65]. This controversy about the role of *Erysipelotrichaceae* in health could be explained by different hosts, cohorts and different bacterial lineages identified. For example, four lineages of *Erysipelotrichaceae* have been identified to respond differently to a single stress [66]. Also, the immunogenic role of *Erysipelotrichaceae* shown in mice was opposed by its lower abundance in cohorts of Crohn’s disease [62].

Finally, an increased abundance was observed in the genus *Bacillus*, which was expected and may be attributable to the test formulation that contains *B. coagulans* as a probiotic. We showed that administration of the formulation every 12 h was sufficient to maintain a stable level of *B. coagulans* among the complex microbiota as confirmed by the qPCR results. The microbiota of rats fed with *B. coagulans* along with standard diet or with an insulin-enriched diet has exhibited an increased abundance of lactic acid bacteria [67]. A previous randomized clinical trial has illustrated that *B. coagulans* could reverse psychological symptoms associated with irritable bowel syndrome such as depression, dementia and sleeplessness [15].

Despite the changes in microbiota composition that suggest a prebiotic effect of the test formulation, *F. prausnitzii* did not exhibit any change with the test treatment. The prebiotic potential of the test formulation was also strongly apparent at the microbiota metabolic capacity. Although no production of GABA, serotonin and dopamine could be detected, treatment of gut microbiome with the test formulation increased the level of butyrate production. Butyrate represents the main energy source for colonocytes, where it induces numerous metabolic and immunological functions [68]. The depletion of major butyrate producers, including *Lactobacillus,* is a common characteristic of IBD microbiota [31,65]. Schulthess et al. have recently shown that preconditioning of macrophages with butyrate induced their antimicrobial effects, which restrict intestinal bacterial growth and increased resistance to entero-pathogens [69]. In addition, the microbiota of young adults with depression were depleted in major butyrate producers and pathways of SCFA generation [70].

## 5. Conclusions

The commercial formulation tested in this study and designed to provide potential prebiotic and psycho-biotic effect effectively showed a synbiotic potential compared to no-treatment and microcrystalline cellulose controls. Mainly, the test formulation had a synbiotic effect on *Lactobacillus* and increased the levels of *Collinsella* and *Erysipelotrichaceae*. These changes in microbiota composition and metabolic profile suggest potential benefits to gut health. Future comprehensive metabolomic and longer-term studies may give more in-depth insights into the microbiota metabolism augmented by the test formulation. However, these results already show that short-term administration of specially formulated ingredients holds the potential for dietary intervention inducing health benefits.

## Supplementary Materials

The following are available online at https://www.mdpi.com/2072-6643/12/9/2669/s1, Figure S1: The composition of gut microbiota developed in the colonic fermentation model during stabilization period. (A) Microbial composition at the family level developed in the inoculating bioreactor over 28 days from immobilized fecal microbiota. The stool samples donated by a third donor (Female, 25 years.). (B) Microbial composition developed in the inoculating bioreactor over the stabilization period for the two donors employed in the current study. Families with abundance >1% are presented while others are grouped together (Others), Figure S2: Relative abundances of gut microbiota Families developed in different bioreactors of colonic fermentation model over 48 h. Samples are named as Donor-Replicate-Bioreactor-Hours post treatment. CR; no treatment control bioreactor, ER1; test formula treated bioreactor, ER2; MC-cellulose treated bioreactor, Figure S3: Cladograms showing the taxonomic relations between significantly differential that are enriched after the test formula treatment as compared to no-treatment control (A) and MC-cellulose control. The analyses and the figure construction were performed by linear discriminant effect size analysis (LEfSe).
