# Supplementary material for: Evaluation of the Prebiotic Potential of a Commercial Synbiotic Food Ingredient on Gut Microbiota in an Ex Vivo Model of the Human Colon
Source: Nutrients. 2020 Sep 1;12(9):2669. doi: 10.3390/nu12092669 (PMC7551822; doi:10.3390/nu12092669)
Supplement: Supplementary file 1 [file nutrients-12-02669-s001.pdf]

Supplementary Materials:

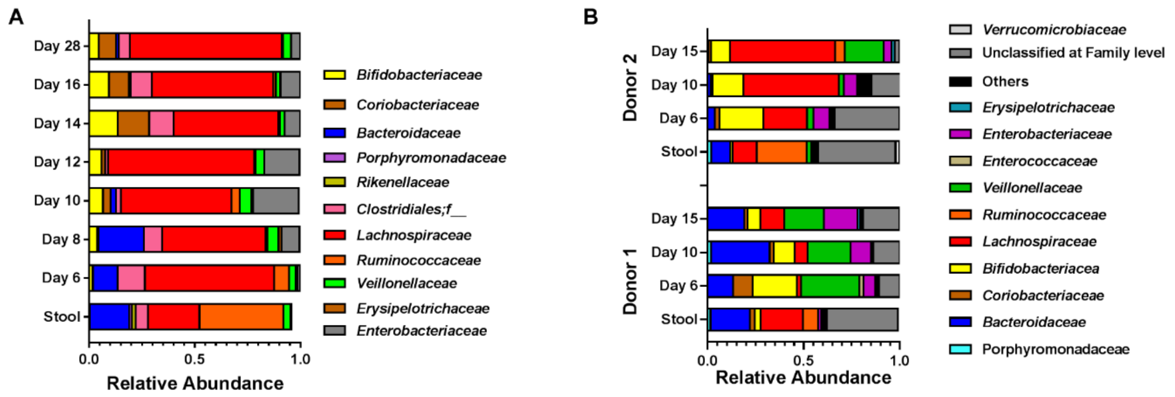

Figure S1. The composition of gut microbiota developed in the colonic fermentation model during stabilization period. A) Microbial composition at the family level developed in the inoculating bioreactor over 28 days from immobilized fecal microbiota. The stool samples donated by a third donor (Female, 25 yrs.). B) Microbial composition developed in the inoculating bioreactor over the stabilization period for the 2 donors employed in the current study. Families with abundance >1% are presented while others are grouped together (Others).

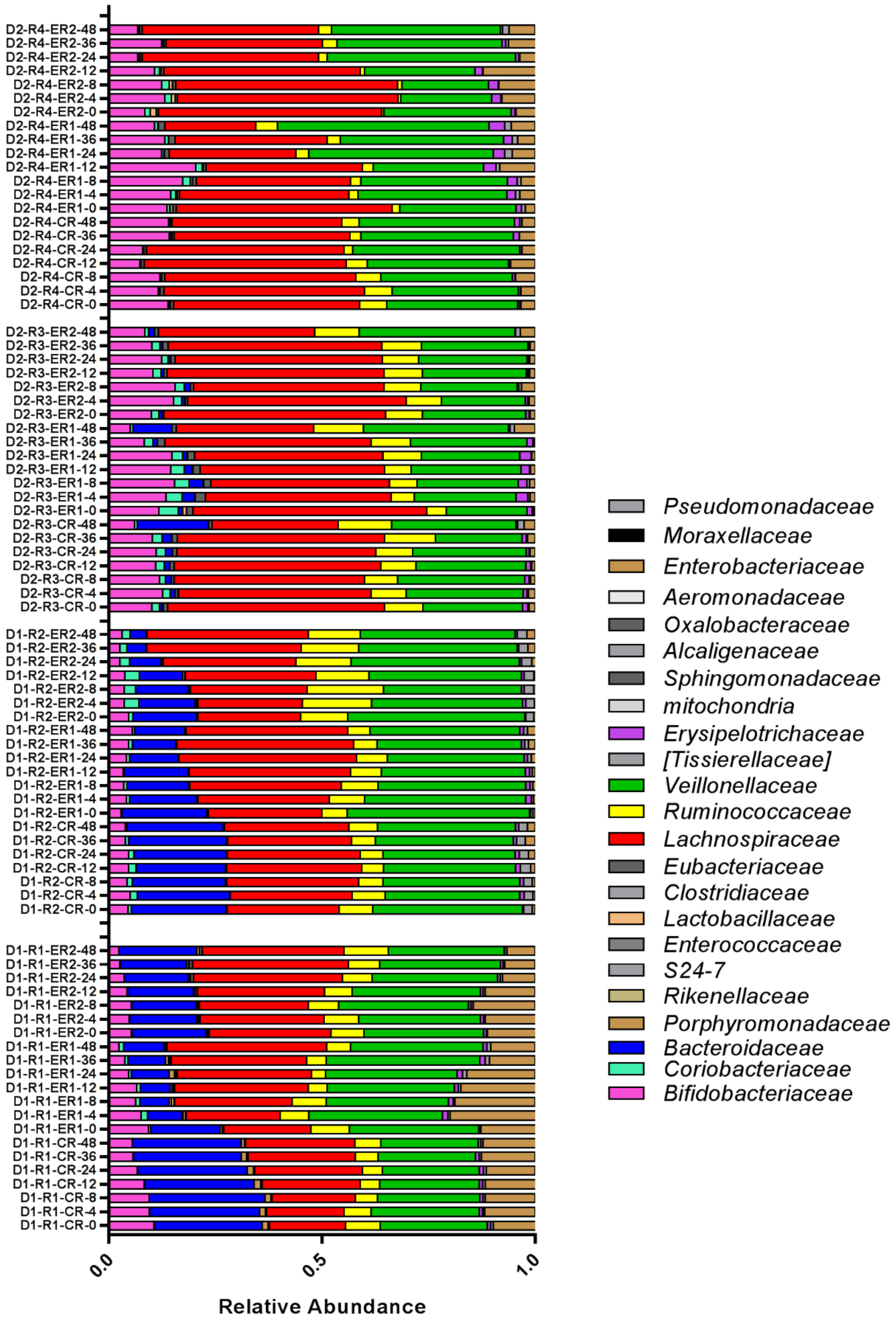

Figure S2. Relative abundances of gut microbiota Families developed in different bioreactors of colonic fermentation model over 48 hours. Samples are named as Donor-Replicate-Bioreactor-Hours post treatment. CR; no treatment control bioreactor, ER1; test formula treated bioreactor, ER2; MC-cellulose treated bioreactor.

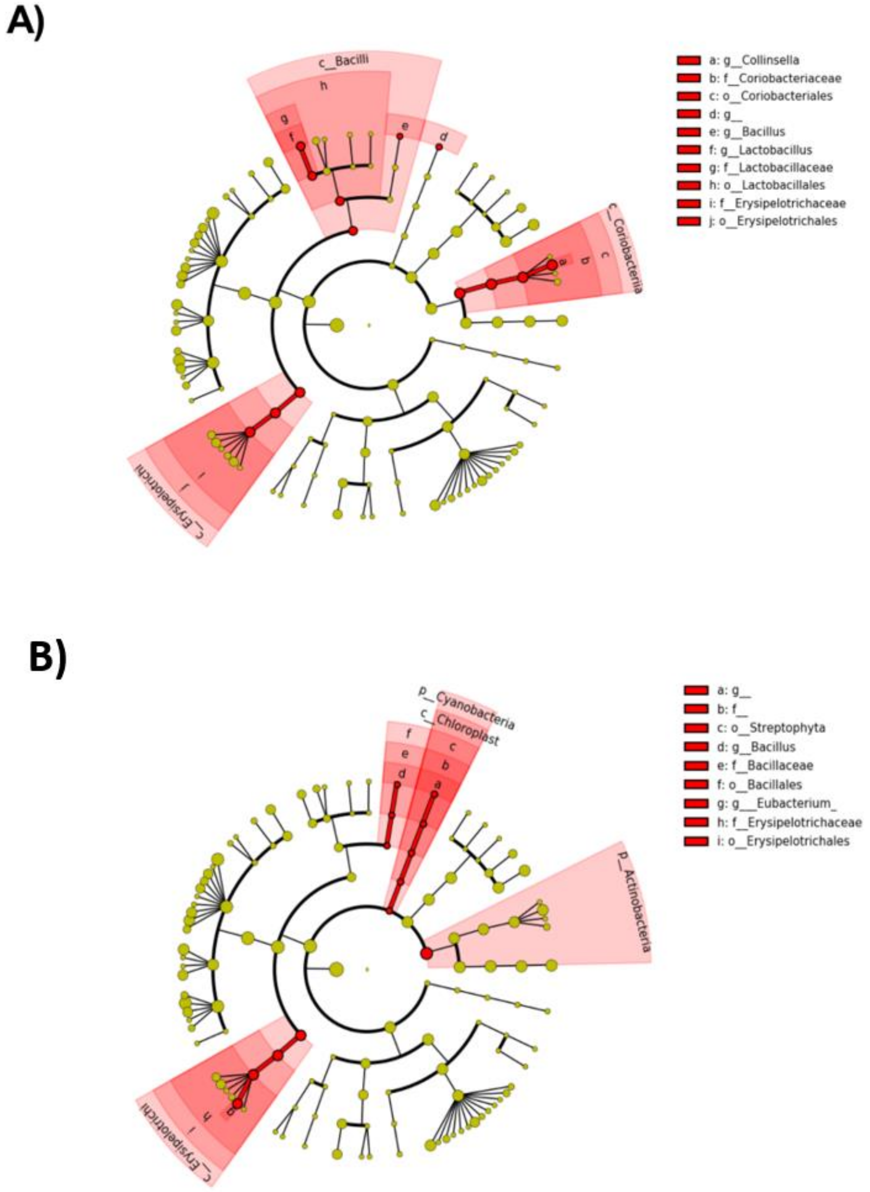

Figure S3: Cladograms showing the taxonomic relations between significantly differential that are enriched after the test formula treatment as compared to no-treatment control(A) and MC-cellulose control. The analyses and the figure construction were done by linear discriminant effect size analysis (LEfSe).
